# Behavioral Change Factors and Retention in Web-Based Interventions for Informal Caregivers of People Living With Dementia: Scoping Review

**DOI:** 10.2196/38595

**Published:** 2022-07-07

**Authors:** Kuan-Ching Wu, Yan Su, Frances Chu, Annie T Chen, Oleg Zaslavsky

**Affiliations:** 1 School of Nursing University of Washington Seattle, WA United States; 2 Department of Biomedical Informatics and Medical Education University of Washington School of Medicine Seattle, WA United States; 3 Biobehavioral Nursing and Health Informatics University of Washington School of Nursing Seattle, WA United States

**Keywords:** dementia, informal caregivers, informal care, caregiving, retention, internet, web-based, behavior, intervention, review, scoping, health intervention, digital health, caregiver, psychological health, cognition, peer support, web-based intervention, taxonomy, aging, gerontology, older adult population, neurological disorder, behavior change technique, BCT, change technique

## Abstract

**Background:**

Web-based interventions aimed at supporting informal caregivers of people living with dementia have the potential to improve caregivers’ well-being and psychological health. However, few interventions are widely implemented for this population, and none of the prior reviews have systematically examined the use of behavior change techniques (BCTs), theories, and agents in web-based interventions for informal caregivers of people living with dementia. To better understand this implementation gap, we reviewed the literature to map behavioral factors (BCTs, theories, and agents) deployed in the studies. Furthermore, because there is an emerging consensus that retention could be shaped by participant characteristics and behavioral factors, we explored relationships between these features and retention rates across studies.

**Objective:**

We pursued 3 objectives: to map behavioral factors involved in the web-based interventions for informal caregivers of people living with dementia; to examine the relationship between behavioral change elements and retention in the studies; and to examine the relationship between participant characteristics (gender, age, and spouse or adult children caregiver proportion) and study retention.

**Methods:**

We conducted a literature review using the following keywords and their corresponding Medical Subject Headings terms: dementia, caregivers, and web-based intervention. The time limits were January 1998 to March 2022. Using the BCTv1 taxonomy, which specifies active behavioral components in interventions, 2 coders collected, summarized, and analyzed the frequency distributions of BCTs. Similarly, they abstracted and analyzed participant characteristics, behavior change theories, behavior change agents, and retention rates in the studies.

**Results:**

The average age was 61.5 (SD 7.4) years, and the average proportion of spousal informal caregivers, adult children informal caregivers, and retention rates were 51.2% (SD 24.8%), 44.8% (SD 22%), and 70.4% (SD 17%), respectively. Only 53% (17/32) of the studies used behavior change theories, but 81% (26/32) included behavior change agents. The most common BCTv1 clusters were *shaping knowledge* and *social support*. The median number of *BCTv1 clusters* was 5 (IQR 3). We observed a negative correlation between the proportion of spousal informal caregivers and the retention rate (*r*=−0.45; *P*=.02) and between the number of BCTv1 clusters and retention rates (*r*=−0.47; *P*=.01). We also found that the proportion of adult children informal caregivers in the study was significantly and positively correlated with the retention rate (*r*=0.5; *P*=.03). No other participant characteristics or behavioral factors were associated with retention rates.

**Conclusions:**

We found that almost half of the studies were not informed by behavior change theories. In addition, spousal involvement and a higher number of BCTs were each associated with lower retention rates, while the involvement of adult children caregivers in the study was associated with higher retention. In planning future studies, researchers should consider matching participant characteristics with their intended intervention as the alignment might improve their retention rates.

## Introduction

### Behavioral Change Interventions for Dementia Caregivers

People living with dementia may have difficulty independently managing their care and typically rely on family and friend caregivers. In fact, 83% of the help provided to older adults with dementia in the United States comes from informal caregivers including family members, friends, or other unpaid caregivers [1]. Given the demand of care involved in dementia, informal caregivers often experience a variety of adverse health complications [1-4]. Compared with caregivers of people living without dementia, informal caregivers of people living with dementia experience 1.5 times higher chances of stroke and a 10% higher occurrence of coronary heart disease, cardiovascular disease, diabetes, and cancer [1,4]. In addition, caregivers of people living with dementia face increased risks of stress, burden, depression, anxiety, and poor quality of life [1-3].

Several behavioral interventions were developed to enhance caregiving knowledge, competencies, and mental health in this population [5-7]. Specifically, cognitive behavioral therapy (CBT) significantly improved depressive symptoms and reduced emotional burden experienced by informal caregivers of people living with dementia [5]. Psychoeducational approaches were also effective in improving caregiving knowledge, well-being, and satisfaction, as well as in reducing caregiver burden, anxiety, and depressive symptoms in caregivers of people living with dementia [6,7]. However, informal caregivers of people living with dementia who are heavily engaged in caregiving tasks or who are at work might not be able to fully participate in the interventions because some behavior programs require face-to-face delivery [8]. Moreover, some specific behavioral interventions are not practical in pandemic settings when in-person contact is discouraged.

### The Use of Web-Based Interventions for Dementia Caregivers

Technology is one method to improve access to care by making psychosocial interventions readily accessible. Web-based interventions, which have been used interchangeably with internet- or web-based interventions, are self-guided or therapist-assisted programs that aim to improve knowledge, provide support, care, or treatment to diverse populations with a range of health problems [9]. Web-based interventions that integrate behavioral change interventions have the ability to incorporate professional and social support, and provide instructions to change behavior and problems in informal caregivers of people living with dementia [10] without requiring face-to-face delivery. Recent studies also indicated that web-based intervention programs can benefit the mental health of caregivers of adults living with chronic conditions, and particularly improve depression, stress and distress, and anxiety in caregivers [8,11]. Thus, web-based interventions aimed at supporting informal caregivers of people living with dementia have the potential to improve their psychological health; however, few interventions are widely implemented for this population [12,13]. A recent review assessing the role and effectiveness of web-based and app-based interventions in the self-management of dementia reported that few studies showed positive outcomes and were effective in improving self-management of people living with dementia [14]. Another meta-analysis that examined the effect of web-based interventions on the mental health outcomes of family caregivers of people living with dementia also found that most internet-based interventions were generally effective in reducing anxiety and depression in caregivers of people living with dementia [13]. However, little research has been done to *look under the hood* concerning the factors relating to behavioral change, such as behavior change theory, behavior change technique (BCT), and behavior change agent (BCA) that informs and shapes the interventions [15,16].

### Behavior Change Theory, BCT, and BCA

In keeping with the definitions provided in Textbox 1, behavior change theories are abstract representations of interrelated concepts, definitions, and propositions that explain behavior change [17]. BCTs are observable, replicable, irreducible, and active ingredients within the intervention designed to change behavior [18]. A BCA is a putative mechanism or process that is measurable and modifiable and is hypothesized to play a causal role in producing behavior change [19]. To date, none of the prior reviews have systematically examined the use of the BCTs and BCAs in web-based interventions for informal caregivers of people living with dementia [12]. More specifically, the extent to which behavior change components are involved in the web-based interventions is still unclear [16]. Moreover, to the best of our knowledge, there is no report to date that explores the relationship of BCTs, BCAs, and retention. Failing to retain a sufficient number of participants in behavior interventions may not only lead to uncertainty about intervention effectiveness and pose a threat to the external validity of the results, but may also be associated with implementation challenges such as increased burden and low engagement [20]. Being able to identify and specify the behavioral active components of web-based interventions and cross-reference them with retention in the studies will provide a better mechanistic understanding of web-based interventions and allow future studies to be replicated more successfully in terms of retention across different settings and populations.

**Definitions for behavior change theory, behavior change technique (BCT), and behavior change agent (BCA).**

**Definitions**

1. Behavior change theory: an abstract representation of an interrelated concept or theory explaining behavior change in the intervention (eg, Stress and Coping model, Cognitive Behavior Therapy Model, Adaption-Coping model, or Transition Theory).
2. BCT: an active, observable, replicable, and intricated component in the intervention which induces the behavior change (eg, goal planning, feedback on behavior, or demonstration of behavior).
3. BCA: a putative target or a mediator variable in the mechanism of behavior change (eg, self-efficacy, caregiver burden, or caregiver stress).

Considering the gaps in behavioral change mechanisms in web-based interventions for informal caregivers of people living with dementia and the increasing number of web-based interventions, it is timely to conduct a scoping review to explore and analyze the emerging literature [21]. This study reviewed the literature to map BCTs, theories, and agents deployed in the web-based interventions for informal caregivers of people living with dementia. Furthermore, there is an emerging consensus that in addition to intervention characteristics, retention could also be shaped by participant characteristics [22-25]. For example, in 2020, Ashford et al [22] identified that sociodemographic variables such as race and education level were associated with decreased task completion and enrollment in web-based interventions for older adults. Another study by Teles et al [25] describing the access and retention in psychosocial interventions for informal caregivers of people living with dementia suggested that caregiver education, their perceived mental health, and the number of hours spent in caregiving had a direct correlation with the retention or dropout rates of the study. As such, this study has three objectives: (1) to map behavioral theories, BCAs, and BCTs involved in web-based interventions for informal caregivers of people living with dementia; (2) to examine the relationship between behavioral change elements and retention in the studies; (3) to examine the relationship between participant characteristics and retention in the studies.

## Methods

### Overview

This scoping review followed guidelines from the PRISMA-ScR (Preferred Reporting Items for Systematic Reviews and Meta-Analyses extension for Scoping Reviews) [26], which was built upon prior scoping review frameworks of the Joanna Briggs Institute [27] and Arksey and O’Malley [28]. The PRISMA-ScR framework deleted 5 items (eg, risk of bias across studies, risk of bias within studies, and further analysis) from the original PRISMA (Preferred Reporting Items for Systematic Reviews and Meta-Analyses) checklist, and it had the following main steps: (1) indicate whether a protocol and registration exist, (2) eligibility criteria, (3) information sources and search, (4) selection of sources of evidence, (5) charting data from the selected studies, and (6) synthesis of results [26].

### Stage 1: Protocol and Registration

The protocol of this paper was modeled on our previous scoping review concerning behavior change factors and retention in dietary interventions for older adults [29]. Our protocol was drafted using the PRISMA Protocols [26]. The final protocol was registered prospectively with the Open Science Framework on February 18, 2022 (registered from the website [30]; registration DOI: 10.17605/OSF.IO/9M7K2).

### Stage 2: Eligibility Criteria

#### Inclusion Criteria

Studies meeting the following inclusion criteria were included: (1) the intervention was aimed at informal caregivers (defined as a family member or friend providing unpaid care) of people living with dementia, (2) digital interventions delivered via the internet or apps, (3) the article considered a specific intervention and provided a description of it, (4) experimental design including quasi-experimental studies (ie, nonequivalent control with pretest-posttest design, nonequivalent control with posttest only, one group pre-post, and time series designs) and randomized controlled trials, (5) feasibility study, (6) published from January 1998 to March 2022, and (7) published in English.

#### Exclusion Criteria

Studies meeting the following exclusion criteria were excluded: (1) studies that focused on people living with early-onset dementia; (2) the intervention was solely delivered by telephone or was telemedicine based; (3) the interventions solely used Skype or other means of web-based calling; (4) the intervention had a large face-to-face component; (5) the results or outcomes of the intervention were not reported; (6) the intervention was focused on the person with dementia; (7) the study was not published in a peer-reviewed journal; (8) basic science articles (eg, animal studies, neuroanatomy, neuroimaging, anatomy, physiology, bacteriology, pathology, or biochemistry) fundamental to the study of medicine; (9) pertained to caregivers aged ≤18 years (per the definition of adults according to the National Institutes of Health); (10) focused on delirium, developmental disorders, or other; (11) letters to the editor, editorials, essays, or other op-ed pieces; (11) gray literature and review articles; (12) other (case study, proposed studies, or study protocol).

We excluded telephone-based support and extensive face-to-face interventions from our study as we intended to focus on digital technologies that could be used by caregivers without professional input.

### Stage 3: Information Sources and Search

A systemic literature search was conducted in 3 databases to identify all relevant literature: PubMed, PsycINFO, and EMBASE. Keywords and Medical Subject Headings (MeSH) terms were used regarding the concepts of mobile health, telehealth, web-based, web, dementia, caregiver. The following were specific keywords used in the searching strategies: (*caregiver* OR *caregivers* OR *carer* OR *carers* OR *Caregivers* [MeSH] OR *care* *partner* OR *care partners*) AND (*Dementia* [MeSH] OR *dementia* OR *dementias*) AND (*“Internet-Based* *Intervention* [MeSH] OR *online* OR *web-based* OR *internet* OR *on-line* OR *electronic* OR *Mobile* *Applications* [MeSH] OR *mobile application* OR *mobile applications* OR *mobile app* OR *mobile apps* OR *tablet* OR *iPad*) AND (*support* OR *supportive* OR *Social Support* [MeSH] OR *Self-Help Groups* [MeSH]). Detailed search strategies are provided in Multimedia Appendix 1. Reference checking was performed to include potentially relevant studies. The research period was from January 1999 to March 2022, considering that the internet was widely used by the general public in 1999.

### Stage 4: Selection of Sources of Evidence

**Figure 1:**
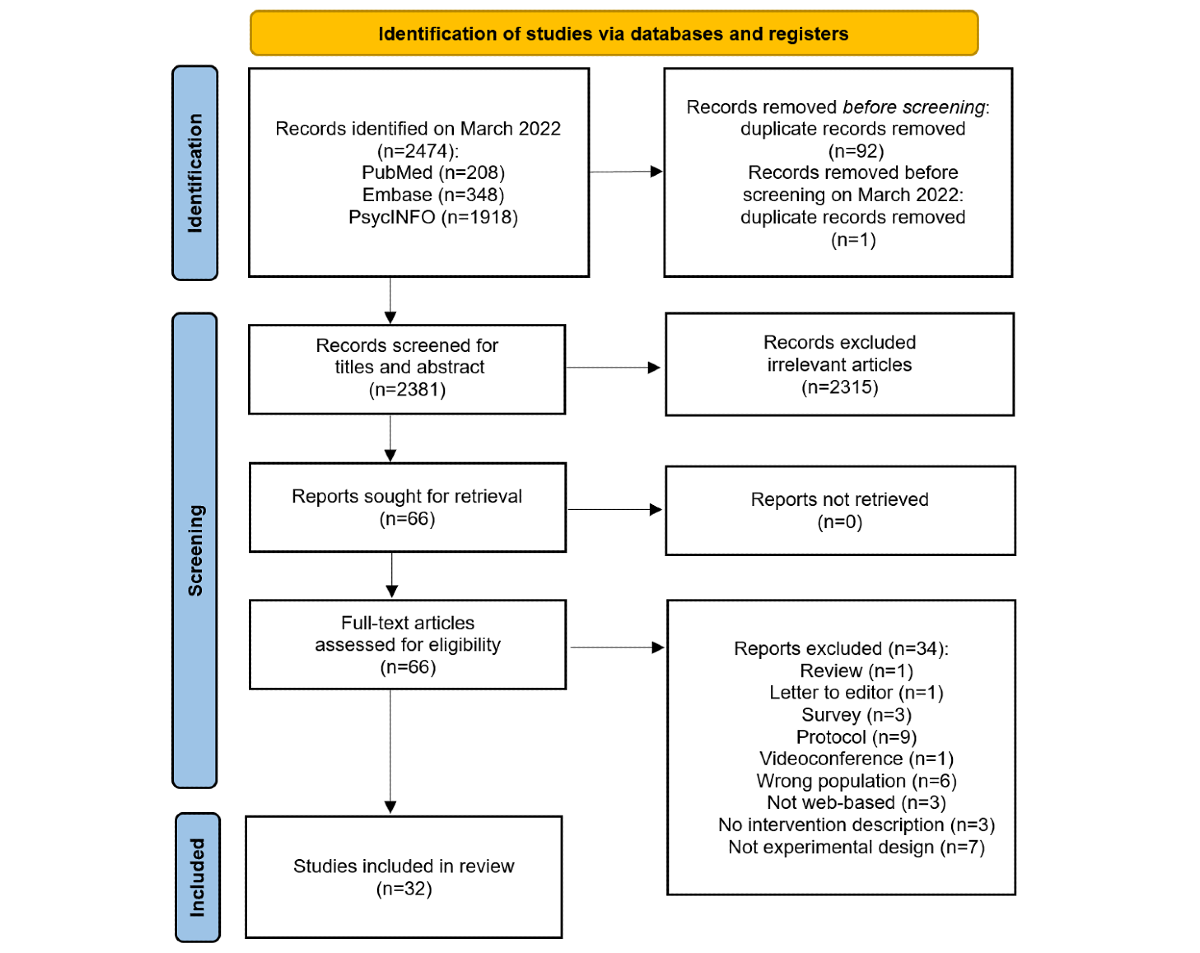
PRISMA-ScR (Preferred Reporting Items for Systematic Reviews and Meta-Analyses extension for Scoping Reviews) flow diagram showing the study selection process.

All citations were uploaded to Rayyan [31], a web-based research tool that helps researchers to collaborate in systemic reviews and other knowledge synthesis projects. Duplicates were removed, and 2 reviewers (K-CW and YS) subsequently screened all the articles by title, abstract, and full text. The reviewers also reviewed each other’s results before proceeding to the next step to avoid screening bias. When a disagreement occurred, they discussed the eligibility of the article regarding the research goal and the inclusion and exclusion criteria until a consensus was reached. In addition, a third reviewer (OZ) was involved to arbitrage disagreements between the 2 reviewers. The detailed screening process is illustrated in Figure 1.

### Stage 5: Charting the Data

After reviewing all eligible studies, 2 reviewers (K-CW and YS) independently coded the literature using the BCTv1 taxonomy [18]. A BCTv1 taxonomy is a taxonomy methodology that comprises 93 individual BCTs grouped in 16 hierarchical clusters (eg, goals and planning, feedback and monitoring, social support, and shaping knowledge) that can specify the active ingredients in interventions. A data-reporting table was generated to guide the data abstraction process and display a summary of these study features: citation, study design, study location, sample characteristic, intervention characteristic, behavior change theory, retention rate, BCT, BCA, and outcome measures.

### Stage 6: Synthesis of Results

We reported results in the following order: (1) sample characteristics; (2) outcomes; (3) theory; (4) BCT; (5) BCA; (6) retention; and (7) relationship among sample characteristics, theory, BCT, BCA, and retention. Sample characteristics included study design, location, caregiver characteristics (type, mean age, and male proportion) and composition. Outcome measures were primary outcomes specified in the objectives and were measured before and after the intervention [32]. Behavior change theories were identified if they were explicitly referenced as the guiding theory or framework for an intervention. When a construct used in the intervention aligned with the behavior change theory or was mentioned in the intervention as a mediator, and was measured before and after the intervention, it would be considered a BCA. To further differentiate BCAs, we categorized each BCA into 3 BCA domains according to the methodology introduced by the Science of Behavior Change [33]. The retention rate was calculated as the percentage of participants who completed the study procedures as prescribed in the protocol. In this study, retention rates were either explicitly stated by the researchers or calculated from flow charts or comparable sources. The relationships between variables and retention rates were assessed using the Spearman rank correlation coefficient because of the observed monotonic but nonlinear trends between the variables, with *P*<.05 indicating statistical significance.

## Results

### Study Selection

The literature review from the PubMed, PsycINFO, and EMBASE databases yielded 2474 results, and 2381 articles were left after duplicates were removed. After abstract and title screening, 2315 irrelevant articles were removed, and 66 studies were retrieved for the full article review. In total, 32 articles were included in the final list after full article eligibility criteria were applied (see Figure 1 for the PRISMA-ScR flow diagram).

### Sample Characteristics

Among the 32 articles, 16 (50%) were conducted in the United States, 10 (31%) were conducted in a European country, 4 (13%) in Canada, 1 (3%) in India, and 1 (3%) in Mexico and South America. In total, 17 studies used a randomized controlled trial in the study design, 8 studies were pilot studies, 4 were feasibility studies, 6 were mixed methods studies, and 3 studies were quasi-experimental. In total, 29 out of the 32 studies included a pretest or posttest design. The reported mean participant age ranged from 44 to 76 years, with a median of 62.4 (IQR 9.52) years and a mean age of 61.48 (SD 7.35) years. The sample sizes ranged from 10 to 486 participants with a median of 63 (IQR 109) participants. The average of reported male proportion was 24.93% (SD 11%; range 0%-53.6%). The mean of the reported spousal and adult children caregiver proportion were 51.24% (SD 25%) and 44.81% (SD 22%), respectively. The main characteristics of the included studies are presented in Multimedia Appendix 2 [34-65].

### Interventions

Almost 80% (25/32) of the interventions were web-based interventions only, while the remaining 20% (7/32) integrated web-based intervention with other telehealth modalities (eg, telephone, virtual reality, email contact, and video conferences); 47% (15/32) of the studies did not include interventionists or facilitators when delivering their interventions. Only 10 studies reported their breakdown by sessions, which ranged from 4 to 8 modules. In addition, 81% (26/32) of the included studies reported the duration of their interventions. Table 1 presents the length of the intervention from the reported studies. The intervention durations varied from 2 weeks to 12 months with a median of 90 (IQR 138) days.

**Table 1:** Intervention period of reported studies (n=32).

Intervention duration	Studies, n (%)
12 weeks	7 (22)
24 weeks	6 (19)
N/A^a^	6 (19)
4 weeks	4 (13)
8 weeks	2 (6)
16 weeks	2 (6)
48 weeks	1 (3)
6 weeks	1 (3)
2 weeks	1 (3)
26 weeks	1 (3)
3 weeks	1 (3)

^a^N/A: not applicable.

### Outcomes

There were at least 18 outcomes measured in different studies. The most common outcome type was the health indicator (25/32, 78%) of informal caregivers, including caregiver burden, stress, depression, pain, and loneliness. The second most measured outcomes were perceived competence (11/32, 34%). Other common outcomes found in the studies were problematic behaviors of care recipients (7/32, 22%), self-efficacy (7/32, 22%), perceived social support (6/32, 19%), quality of life (5/32, 16%), caregiving knowledge (4/32, 13%), quality of the relationship with care recipients (4/32, 13%), perceived health (4/32, 13%), and intervention usability and feasibility (4/32, 13%). Some peculiar outcomes include eHealth literacy, heart rate variability, costing, and cost-effectiveness.

### Theories

In total, 53% (17/32) of the studies explicitly mentioned using behavior change theories or models to guide their interventions. Of these, only 4 studies mentioned more than one model. As Table 2 shows, the most used theory was the Stress and coping theory (6/17, 35%) and the CBT (6/17, 35%). Other theories or models were psychoeducational intervention (5/17, 29%), Transition theory (2/17, 12%), Trigger behavior response (1/17, 6%), Adaption-coping model (1/17, 6%), Social cognitive theory (1/17, 6%), Stress process model (1/17, 6%), and Communities of practice theory (1/17, 6%).

**Table 2:** Theories specified in guiding the interventions (n=32). An intervention could be guided by more than one theory.

Theory	Studies, n (%)
N/A^a^	15 (88)
Stress and coping model	6 (35)
Cognitive behavioral therapy	6 (35)
Psychoeducation	5 (29)
Transition theory	2 (12)
Trigger behavior response	1 (6)
Stress process model	1 (6)
Adaption-coping model	1 (6)
Social cognitive theory	1 (6)
Communities of practice theory	1 (6)

^a^N/A: not applicable.

### BCAs and BCA Domains

We found BCAs in 81% (26/32) of the studies. The most common BCA was caregiver burden or strain (14/26, 54%). Other common BCAs included self-efficacy or confidence (10/26, 38%), caregiver stress or distress (10/26, 38%), social support (8/26, 31%), and caregiving competence or skill mastery (8/26, 31%). In addition, using the approach adopted from Nielsen et al [19] and the Science of Behavior Change taxonomy, which clusters BCAs into 3 major groups (interpersonal, stress reactivity, and self-regulation), we found that *stress reactivity* (20/26, 77%) was the most common BCA featured in 16 studies. The second commonly used BCA was *self-regulation* (18/26, 69%), and the least common was *interpersonal* (11/26, 42%).

### Behavior Change Techniques

All 32 articles included at least one BCT, and 97% (31/32) of the studies included at least 2 BCTs in their intervention. The total number of individual BCT included in the studies ranged from 1 to 14, with a median of 5 (IQR 4) techniques. The total number of BCTv1 clusters ranged from 1 to 9 with a median of 4.5 (IQR 3) clusters. The individual BCT in each study is listed in Multimedia Appendix 3, and the frequency distribution of BCTv1 clusters specified in each study is presented in Table 3. The most frequently deployed BCTv1 cluster was *shaping knowledge* (27/32, 84%). The other common clusters include *social support* (19/32, 59%), *comparison of outcomes* (19/32, 59%), *comparison of behavior* (18/32, 56%), and *goals and planning* (17/32, 53%).

**Table 3:** Behavior change techniques (BCTs) taxonomy specified in guiding the interventions. One BCT cluster might appear in multiple studies (n=32).

BCT cluster	Studies, n (%)
1. Goals and planning	17 (53)
2. Feedback and monitoring	13 (41)
3. Social support	19 (59)
4. Shaping knowledge	27 (84)
5. Natural consequences	3 (9)
6. Comparison of behaviors	18 (56)
7. Associations	4 (13)
8. Repetition and substitution	4 (13)
9. Comparison of outcomes	19 (59)
10. Reward and threat	1 (3)
11. Regulation	1 (3)
12. Antecedents	10 (31)
13. Identity	10 (31)
14. Scheduled consequences	0 (0)
15. Self-belief	4 (13)
16. Covert learning	0 (0)

### Retention

Retention rates were extracted from 91% (29/32) of the studies. The range of the retention rates from included studies varied from 32.6% to 97.4%, with an average of 70.44% (SD 17%) and a median of 74.6% (IQR 15%). Considering 80% as the third quantile of the retention rate in the included studies, we defined any study with an 80% retention or above as a high retention rate study. Only 28% (9/32) [34,41,44,48,49,52,53,55,63] of the interventions were high retention rate studies.

### Retention With BCA, BCT, and Sample Characteristics

**Figure 2:**
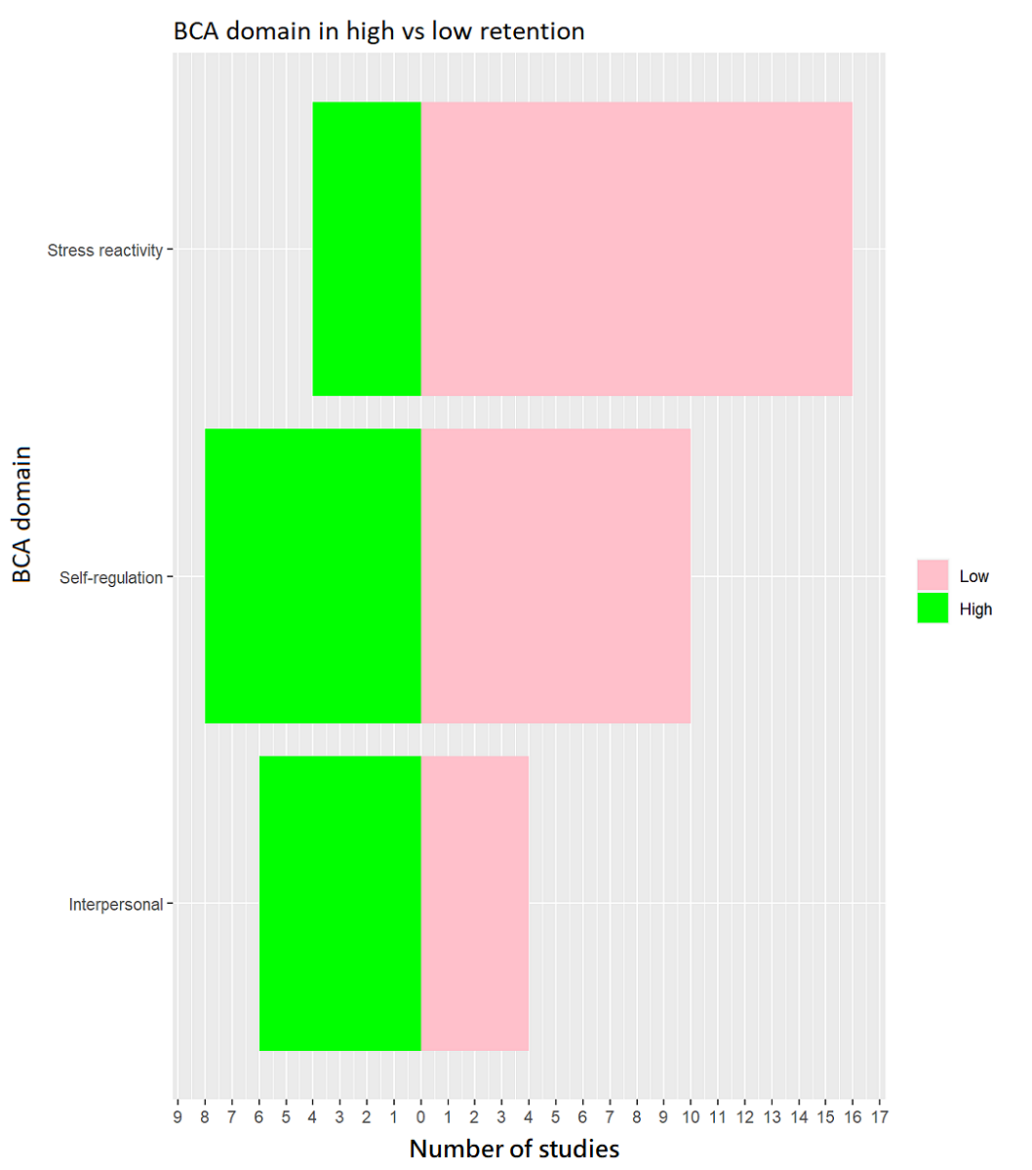
Behavior change agent (BCA) domains used in high vs low retention studies.

As shown in Figure 2, when examining high or low retention studies by 3 BCA domains (stress reactivity, self-regulation, and interpersonal), we found that stress reactivity was more common in low retention studies (n=16) while self-regulation (n=8) and interpersonal (n=6) were more common in high retention studies.

**Figure 3:**
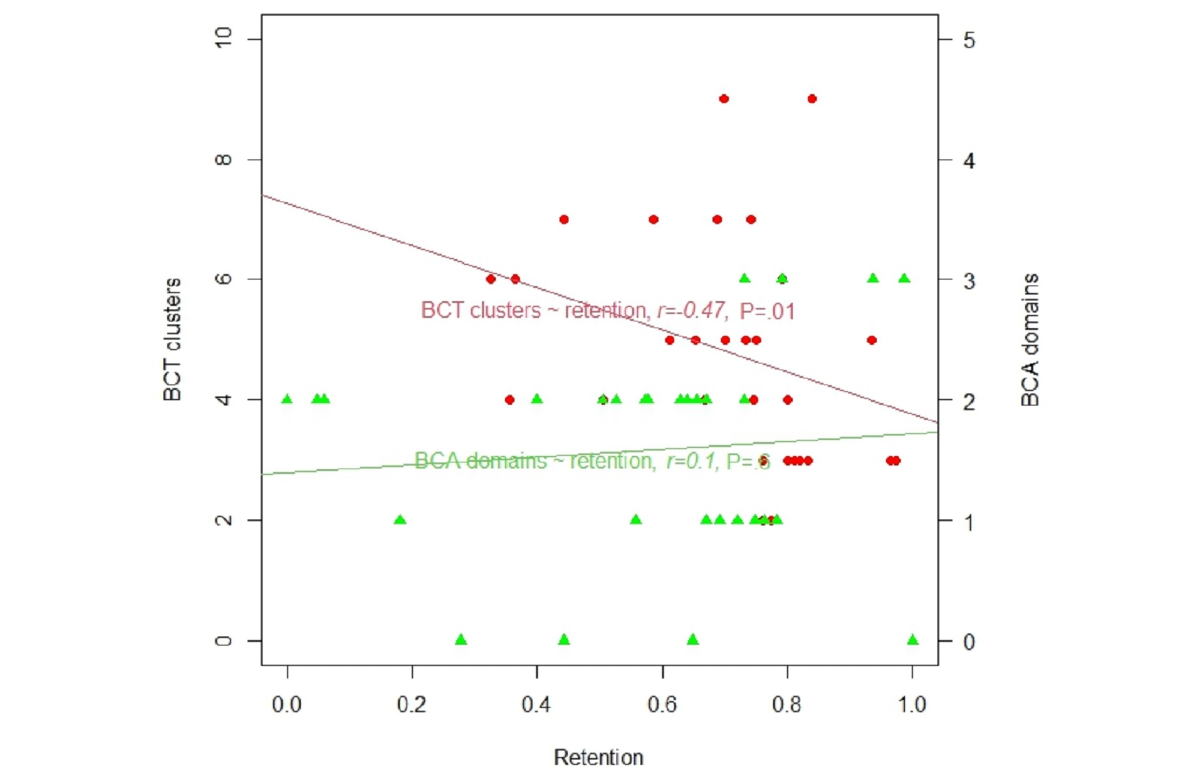
Relationships of retention rate to behavior change technique (BCT) clusters and behavior change agent (BCA) domains.

Figure 3 shows the relationships between retention rate and BCTv1 clusters or BCA domains. The Spearman coefficient suggested no significant relationship between the retention rates and the BCA domains (*r*=0.1; *P*=.60). However, there was a significant and negative relationship between the retention rate and the number of BCTv1 clusters (*r*=−0.47; *P*=.01).

**Figure 4:**
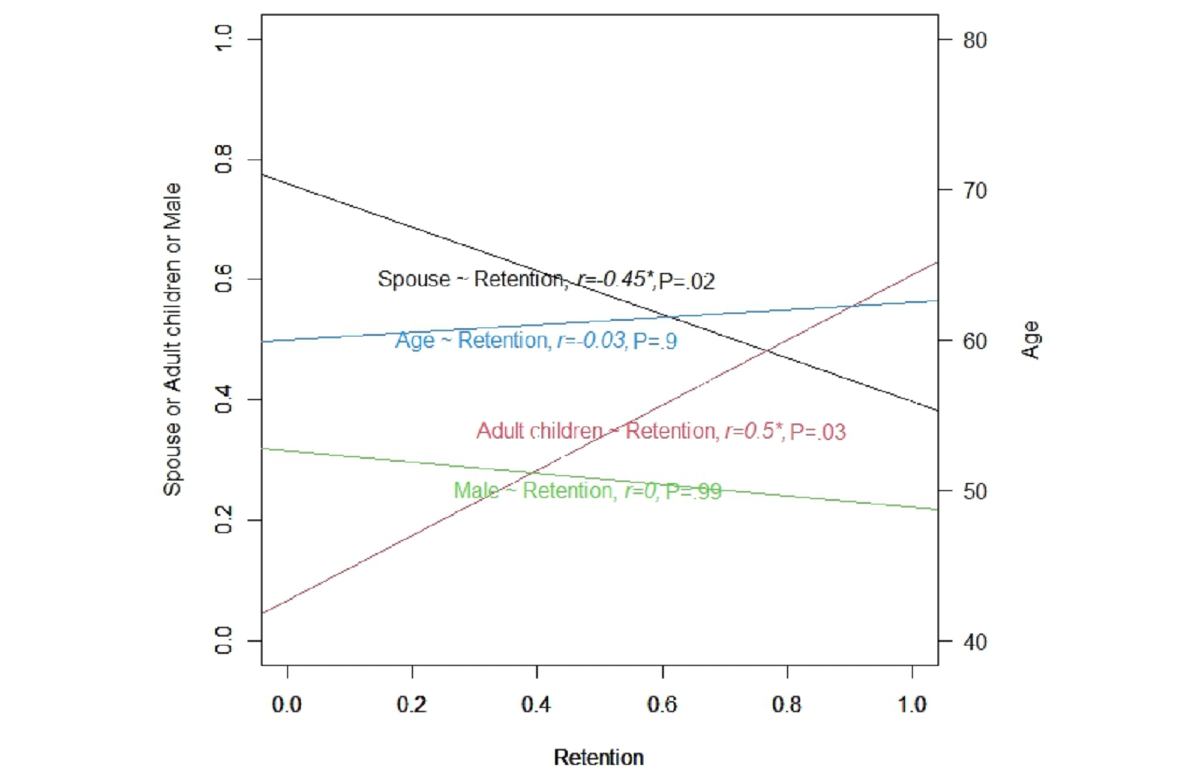
Relationships of retention rate to age, gender, spouse, and adult children proportion. *: *P*<.05.

Figure 4 presents the relationships of retention rate to age, gender, and spouse or adult children proportion. According to the Spearman correlation coefficient, no significant differences were found between the retention rate and informal caregiver’s age (*r*=−0.03; *P*=.90) and gender (*r*=0; *P*=.99). However, we found that the proportion of spousal caregivers was significantly and negatively correlated with the retention rate (*r*=−0.45; *P*=.02), whereas the proportion of the adult children caregivers was significantly and positively correlated with the retention rate (*r*=0.5; *P*=.03) in the studies.

## Discussion

### Principal Findings

The goals of this scoping review were to describe the level of evidence of behavioral factors (theory, BCA, and BCT) in web-based interventions for the informal caregivers of people living with dementia and to examine the relationship between sample characteristics, behavioral change factors, and study retention rates. We have 3 major findings. First, only about a half of the studies described their theoretical framework, but BCTs and BCA were more common. Second, we found that average retention rate has been around 70%, suggesting that it is a challenge for most web-based interventions in this population to retain participants. Third, the number of BCTv1 clusters and proportion of spousal caregivers are significantly and negatively correlated with retention rates while the involvement of adult children caregivers is significantly and positively correlated with the retention rate in the studies.

### Comparison With Prior Work

The first finding indicates that about half of the web-based interventions lacked theory to guide their intervention. This finding is congruent with the results of 3 systematic reviews of health interventions in people with chronic conditions. In 2017, a review of exercise interventions in people living with dementia found 33% included studies used behavior change theories [66]. A review in 2019 of interventions targeting people with chronic neurological conditions and their caregivers found 59% of the studies did not mention theory, and only 22% were explicitly theory-based [67]. Another review of interventions among dementia caregivers revealed that only 37.5% of the studies used theory to inform interventions [68]. As mentioned by Walsh et al [15], there is a distinct lack of theoretical underpinnings in most dementia interventions [15], and interventions that make extensive use of theory may be able to have larger effects on behavior and improve the intervention sustainability than those that lack theory [15,69]. The explicit application of theory can help a study to better understand key aspects of the intervention, the participants, and the context, and offer a generalizable framework to inform the development of intervention as well as provide insights to possible causal mechanisms [15,70]. Therefore, we strongly advocate future research in the dementia caregiving context to include a theoretical basis in their intervention design.

In this study, we found that stress and coping theory, CBT, and psychoeducation are the most commonly adopted theories among web-based interventions for informal caregivers of people living with dementia. This finding partially aligns with several studies which specified CBT and psychoeducational approaches as the most effective and common theories in caregiver interventions which aimed to improve caregiving knowledge, well-being, and satisfaction as well as to reduce caregiver burden, anxiety, and depressive symptoms in caregivers of people living with dementia [5-7]. However, these reviews focused less on web-based interventions and may not provide the full picture of how theoretical underpinnings could guide interventions delivered on the web or in web-based settings. On the contrary, a meta-analysis released in 2021, which explored how web-based interventions improve mental health in home caregivers of people living with dementia, provided a good rationale for our finding in the *stress and coping theory*. The meta-analysis found that stress management program showed better outcomes in web-based interventions than other modified multicomponent integration programs [14]. Therefore, we suggest that future web-based intervention studies should retain the systemic integrity of the stress coping model when building their interventions.

In our review, 80% of the studies included BCA and all the studies included at least one BCT in their intervention, but the number of BCTs varied substantially. A recent review which assessed BCTs in clinical interventions confirmed their effectiveness in retention context [16] that is in sync with our results. Furthermore, both studies described a range BCTs in terms of numbers guiding their intervention design. For example, Fakolade et al [67] found that, *“*across 27 studies, two to 17 BCTs (mean 6.8, SD 4.02) were used.” We found the top three frequently deployed BCTv1 clusters were *shaping knowledge*, *social support*, and *comparison of outcomes*. These results are similar to a systematic review of internet-based interventions for caregivers of older adults [71]. This systematic review found the most frequently used BCTs included in efficacious interventions were provision of social support and the combination of instructions to guide behavior change and barrier identification. Another systematic review which mapped behavioral factors in health interventions for people with chronic neurological conditions and their caregivers shared analogous views that *shaping knowledge* and *comparison of outcomes* are 2 of the most common implemented BCT clusters [67]. To date, no reviews have mapped behavioral change factors with web-based interventions in dementia research. However, 2 systematic reviews that evaluated web-based self-management programs for parental caregivers to help children with diabetes and promote healthy eating in children both confirmed that *shaping knowledge* is a widely used and effective BCT cluster in web-based program interventions [72,73]. Future research in web-based interventions for dementia caregivers should consider retaining *shaping knowledge* while developing their interventions. Unfortunately, we did not find reviews which targeted interventions for people living with dementia and their caregivers stipulating BCA in their study. Considering increasing calls from the National Institutes of Health to emphasize on mechanisms of change [19], BCA should be explicitly specified and evaluated in future intervention development.

Our second major finding is that retention is still a challenge in most web-based interventions for caregivers of people living with dementia. We calculated an average of 70.44% (SD 17%) and a median of 74.6% (IQR 15%) retention rate in 32 studies. This result is similar to a cross-sectional study of retention of dementia caregivers in psychosocial interventions which reported high dropout rates (more than 50% for most intervention) in most psychosocial interventions [25]. Nevertheless, compared with the study by Teles et al [25], it seems the web-based interventions increased retention among this population. Our result about low retention rate contrasted with a systematic review which examines the effectiveness of mobile and web-based health apps that support self-management and transition in young people with chronic physical health illnesses. The review reported an average 93% retention across 68 studies. The reasons that this systematic review observed higher retention compared with our study might result from the differences in our target populations (young people with chronic disease versus older adult dementia caregivers). Older adult participants may face more barriers in web-based interventions compared with younger generations owing to age-related changes (eg, changes in vision, hearing, and motor functions) [74]. Therefore, we might expect a lower retention in older participants in web-based interventions, especially in self-guided web-based interventions [75,76]. Future web-based interventions for informal caregivers of people living with dementia should consider the needs of this population and incorporate them in the development of the interventions.

The third major finding in our study is that retention rate was significantly associated with certain behavioral factors and sample characteristic. We identified a reduction in retention with an increase of BCTv1 clusters employed in interventions. The finding that the numbers of BCTv1 clusters is negatively associated with retention is at odds with the systematic review conducted by Duncan et al [16], which advocates for the application of BCTs to improve retention. Another meta-analysis which investigated how incorporating BCTs in internet-based programs could reduce smoking cessation in the public also presents different evidence from what was found in the study [77]. The meta-analysis reported that the number of BCTs in the long term was not significantly associated with treatment effectiveness (odds ratio 1.02, 95% CI 0.99-1.05; *P*=.16). However, the meta-analysis did not correlate the BCT numbers and BCTv1 clusters with retention rates. To conclude, our study raises several important issues for future research. For example, it would be important to examine the extent to which BCT potentially affect retention. If such an effect confirmed, factors facilitating retention rate such as particular BCTv1 clusters, the overall number of BCTv1 clusters or other features is another important consideration. Future studies should work on better elucidating the mechanisms of behavioral change, and explore how behavioral factors (theories, BCTs, and BCAs) affect the effectiveness and retention of the intervention, as well as provide more details on how BCTs should be leveraged.

As for sample characteristics, we found that the increase of spousal caregivers or the decrease of adult children caregivers in the study are significantly correlated with lower retentions. There were several studies that considered sample characteristics as predictors of retention. For example, a meta-analysis which identified predictors of treatment dropout in self-guided web-based interventions for depression found that being male, younger age, attained lower education level, and with comorbid anxiety symptoms were all predictors for a high dropout rate [75]. Some other studies also reported being young [23,24], less educated [22-24], race of people of color [22,23], or person with a lower socioeconomic status [24] were factors associated with lower retention rate in the intervention. A study of the access and retention of informal dementia caregivers in psychosocial interventions also reported significant associations between retention rate and behavioral factors of caregivers such as the number of hours spent in caregiving, and informational barriers [25]. However, we did not identify any study that associated retention rate with the ratio of spousal caregivers or adult children caregivers.

### Limitations

Several limitations should be considered in this scoping review. One limitation was a lack of clarity in some interventions and theories. In some of the studies, interventions and the theoretical framework were not fully specified. Poorly justified intervention design and lack of detail descriptions of intervention could lead to challenges for researchers to abstract the BCTs or affect the analysis of behavioral factors in each intervention. Another limitation of this scoping review is the lack of other demographics information such as ethnicity, socioeconomic status, and education levels of caregivers. These are not available in many included studies but they might affect findings about adult children and spouse relationship with retention. A final limitation is the inconsistency in the intervention duration, which is the length of each web-based intervention. The length of the interventions ranged from 3 weeks to 12 months, which might ultimately impact the retention rate of the study. It is our recommendation for future studies to consider the length of the interventions and their effects on the retention rates.

It is worth mentioning that the sample characteristics of our review might not be generalizable to the older informal caregivers of people living with dementia (caregivers aged ≥65 years), because the reported mean age of informal caregivers of people living with dementia in the studies reviewed was 8 years younger compared with the mean age provided by the American Association of Retired Persons (AARP) and Alzheimer’s Association. According to the Alzheimer’s Association and AARP, women account for approximately two-third of dementia caregivers, with an average age of 69.4 years [1,78], while the reported mean age of informal caregivers of people living with dementia in our study was 62.26 (SD 7.36) years. Possible reasons that the mean age of the studies our sample were younger than the actual dementia caregiver population reported by the AARP and Alzheimer’s Association are that web-based interventions may create specific challenges for the older populations to participate in the study [79,80]. For example, limited access to high-speed internet and video chatting, owning older technology that induces hardware and software incompatibilities, unfamiliarity with new technologies and motivational barriers, or visual impairments that affect the comprehension of interventions are all possible factors that reduce the participation of older adult dementia caregivers. Although Alzheimer’s Association announced that over half of the caregivers are providing assistance to a parent or an in-law with dementia [1], they did not specify the proportions of spousal caregivers and adult children caregivers across the informal caregiver population. Our study, however, found that the means of the reported spousal caregiver and adult children caregiver proportions in 32 studies were both around 50% (51% and 45% individually), with the reported spousal caregiver proportion being 6% more than the adult children caregiver proportion.

### Conclusions and Future Implication

This is the first study to comprehensively describe behavioral change factors (theory, BCA, and BCT) and identify the active ingredients in web-based interventions for the informal caregivers of people living with dementia using the BCTv1 taxonomy. This is also the first study to map the relationship of retention rates with behavioral change factors and sample characteristics.

In our study, we found that almost half of the studies were not informed by behavior change theories. We also observed that web-based interventions for informal caregivers of people living with dementia usually face retention challenges. Furthermore, we found that spousal involvement and a higher number of BCTs were each associated with lower retention rates.

On the basis of these findings, we proposed 3 suggestions for future studies. In planning future web-based interventions for informal caregivers of people living with dementia, researchers should (1) report the theoretical basis and behavioral change factors informing their study design; (2) ensure to address the needs of informal caregivers and people living with dementia in intervention development; and (3) model mechanisms of behavioral change and further explore how behavioral factors (theories, BCTs, and BCAs) and sample characteristics affect the effectiveness and retention of the intervention, as well as provide more details on how BCTs are applied.

## Appendix

Multimedia Appendix 1 Key literature search terms and search results.

Multimedia Appendix 2 Characteristics of included studies.

Multimedia Appendix 3 The behavioral change factors (theory, techniques, and agents) identified in each study.

## Abbreviations

AARP: American Association of Retired Persons
BCA: behavior change agent
BCT: behavior change technique
CBT: cognitive behavioral therapy
MeSH: Medical Subject Headings
PRISMA: Preferred Reporting Items for Systematic Reviews and Meta-Analyses
PRISMA-ScR: Preferred Reporting Items for Systematic Reviews and Meta-Analyses extension for Scoping Reviews
