# Supplementary material for: Behavioral Change Factors and Retention in Web-Based Interventions for Informal Caregivers of People Living With Dementia: Scoping Review
Source: J Med Internet Res. 2022 Jul 7;24(7):e38595. doi: 10.2196/38595 (PMC9305400; doi:10.2196/38595)
Supplement: Multimedia Appendix 1 [file jmir_v24i7e38595_app1.pdf]

Databases	Terms	Results (*n*)
PubMed	("Caregivers"[Mesh] OR caregiver OR caregivers OR carer OR carers OR "care partner" OR "care partners") AND ("Dementia"[Mesh] OR dementia OR dementias) AND (("Internet-Based Intervention"[Mesh] OR "Mobile Applications"[Mesh] OR "Computers, Handheld"[Mesh] OR digital OR online OR web-based OR internet OR on-line OR electronic OR "mobile application" OR "mobile applications" OR "mobile app" OR "mobile apps" OR tablet OR tablets OR iPad OR mHealth) AND ("Social Support"[Mesh] OR "Self-Help Groups"[Mesh] OR support OR supportive))<br>Filters: Clinical Study, Clinical Trial, Clinical Trial Protocol, Clinical Trial, Phase I, Clinical Trial, Phase II, Clinical Trial, Phase III, Clinical Trial, Phase IV, Comparative Study, Controlled Clinical Trial, Evaluation Study, Interview, Multicenter Study, Observational Study, Pragmatic Clinical Trial, Randomized Controlled Trial, Twin Study, Validation Study, Humans, English, from 1998 - 2021	208
Embase	#1 'caregiver'/exp OR caregiver* OR carer* OR 'care partner' OR 'care partners'<br>#2 'dementia'/exp OR dementia*<br>#3 'web-based intervention'/exp OR 'mobile application'/exp OR 'handheld device'/exp OR 'mobile phone'/exp OR digital OR online OR 'web based' OR internet OR 'on line' OR electronic OR 'mobile application' OR 'mobile applications' OR 'mobile app' OR 'mobile apps' OR tablet* OR ipad OR mHealth<br>#4 'caregiver support'/exp OR 'social support'/exp OR 'self care'/exp OR support*<br>#5 #3 AND #4<br>#6 #1 AND #2 AND #5<br>Limits<br>AND [embase]/lim NOT ([embase]/lim AND [medline]/lim) AND ('clinical article'/de OR 'clinical protocol'/de OR 'clinical trial'/de OR 'cohort analysis'/de OR 'comparative effectiveness'/de OR 'comparative study'/de OR 'controlled clinical trial'/de OR 'controlled study'/de OR 'cross sectional study'/de OR 'exploratory research'/de OR 'feasibility study'/de OR 'grounded theory'/de OR 'human'/de OR 'human experiment'/de OR 'intervention study'/de OR 'interview'/de OR 'longitudinal study'/de OR 'major clinical study'/de OR 'medical record review'/de OR 'methodology'/de OR 'model'/de OR 'multicenter study'/de OR 'normal human'/de OR 'observational study'/de OR 'pilot study'/de OR 'prospective study'/de OR 'qualitative research'/de OR 'quality control'/de OR 'questionnaire'/de OR 'randomized controlled trial'/de OR 'retrospective study'/de OR 'sample size'/de OR 'semi structured interview'/de OR 'single blind procedure'/de OR 'statistical model'/de OR 'telephone interview'/de OR 'theoretical study'/de OR 'total quality management'/de OR 'validation process'/de OR 'validation study'/de) AND 'article'/it	348
PsycINFO	#1 DE "Caregivers" OR DE "Caregiving" OR caregiver* OR carer* OR "care partner" OR "care partners"<br>#2 DE "Dementia" OR DE "AIDS Dementia Complex" OR DE "Dementia with Lewy Bodies" OR DE "Presenile Dementia" OR DE "Pseudodementia" OR DE "Semantic Dementia" OR DE "Senile Dementia" OR DE "Vascular Dementia" OR dementia*<br>#3 DE "Online Social Networks" OR DE "Digital Interventions" OR DE "Online Therapy" OR DE "Mobile Technology" OR DE "Mobile Applications" OR DE "Wearable Devices" OR DE "Mobile Devices" OR DE "Mobile Phones" OR DE "Tablet Computers" OR DE "Mobile Health" OR digital OR online OR web-based	1918

OR internet OR on-line OR electronic OR "mobile application" OR "mobile applications" OR "mobile app" OR "mobile apps" OR tablet* OR iPad OR mHealth

#4 DE "Social Support" OR DE "Support Groups" OR DE "Self-Help Techniques" OR support*

#5 #3 AND #4

#6 #1 AND #2 AND #5

Limiters - Scholarly (Peer Reviewed) Journals; Publication Year: 1998-2021; Language: English; Population Group: Human; Methodology: CLINICAL CASE STUDY, CLINICAL TRIAL, EMPIRICAL STUDY, -Experimental Replication, -Followup Study, -Longitudinal Study, ---Prospective Study, ---Retrospective Study, FIELD STUDY, INTERVIEW, -Focus Group, QUALITATIVE STUDY, QUANTITATIVE STUDY, TREATMENT OUTCOME, TWIN STUDY; Exclude Dissertations
