# Supplementary material for: Behavioral Change Factors and Retention in Web-Based Interventions for Informal Caregivers of People Living With Dementia: Scoping Review
Source: J Med Internet Res. 2022 Jul 7;24(7):e38595. doi: 10.2196/38595 (PMC9305400; doi:10.2196/38595)
Supplement: Multimedia Appendix 2 [file jmir_v24i7e38595_app2.pdf]

Multimedia Appendix 2-Characteristics of included studies. ICG: Informal Caregiver; FCG: Family Caregiver; PLWD: Persons Living with Dementia; ADRD: Alzheimer's Disease and Related Dementias.

Study, Design, Location	Sample (participants, mean age, male proportion, ICG composition)	Intervention Duration	Retention	Outcomes
Shaw et al. (2021) [1],<br>multi-site RCT; pre-post,<br>USA	N=68, mean age 65.03,<br>27.9% male,<br>64.7% spouse; 33.8% children; 1.5% Others	3 months	NA	Process-based costing, cost-effectiveness analysis, caregiver depression, caregiver competence
Williams et al. (2021) [2],<br>mixed-method feasibility study with multisite RCT; pre-post,<br>USA	N=83, mean age 64.2,<br>28.9% male,<br>66.3% spouse; 32.5% children; 1.2% Others	3 months	50.60%	Quantitative: satisfaction, utilization<br>Qualitative: feasibility and future adaptations
Bruinsma et al. (2021) [3],<br>feasibility RCT; pre-post,<br>Netherlands	N=27, mean age 64.5,<br>11.1% male,<br>100% spouse	NA	74.10%	Self-efficacy, caregiver mastery, Self-perceived stress, anxiety
Baruah et al. (2021) [4],<br>RCT; pre-post,<br>India	N=151, mean age 44.3,<br>53.6% male,<br>20.53% spouse; 79.47% non-spouse	3 months	36.42%	Primary: burden, depression;<br>Secondary: person-centered attitude, self-efficacy, mastery and self-rated health
James et al. (2021) [5],<br>pilot RCT; pre-post,<br>USA	N=28, mean age 60.3,<br>26.3% male,<br>63.2% spouse; 31.6% children; 5.3% Others	2 weeks	35.71%	Quality of life, self-compassion, Heart Rate Variability, caregiver burden, anxiety
Fossey et al. (2021) [6],<br>three-armed parallel-group RCT; pre-post,<br>USA	N=638, mean age 59.9,<br>15% male,<br>44% spouse; 37% children; 19% Others	26 weeks	32.60%	Mental health, anxiety, depression, overall mood, caregiving stress, caregiver mastery
Romero-Mas et al., (2021) [7],<br>quasi-experimental; pre-post,<br>Spain	N=38, mean age 55.73,<br>21% male,<br>13.16% spouse; 78.94% children; 7.89% Others	NA	97.40%	Caregiver's quality of life, eHealth literacy
Verkade et al. (2020) [8],<br>3-arm RCT; pre-post;<br>Netherlands	N=81, mean age 75.1,<br>12% male,<br>40% spouse; 57% children; 3% Others	NA	82.00%	Self-efficacy,<br>caregiver reports of behavior problems in PLWD, the quality of the relationship
Gustafson Jr et al. (2019) [9],<br>RCT; pre-post,<br>USA	N= 31, mean age not mentioned (81% are above 65 year-old),<br>39% male,<br>90% spouse; 3% children; 7% Others	6 months	83.90%	Caregiver burden, family conflict, satisfaction with decisions, social support, loneliness, anxiety, depression, coping competence
Duggleby et al. (2018) [10],<br>multisite; mixed methods; longitudinal; RCT; pre-post,<br>Canada	N=199, mean age 63.6,<br>19% male,<br>49% spouse or life-time partner; 46% children; 5% other	3 months	77.38%	Health-related quality of life (HRQOL), self-efficacy, hope in caregivers
Duggleby et al. (2018) [11],<br>mixed-methods; single-arm; pre-post,<br>Canada	N=37, mean age 63.24,<br>35% male,<br>29.7% spouse; 59.5% children; 10.8% Others	2 months	81.08%	hope, general self-efficacy, loss and grief , and health-related quality of life

Study, Design, Location	Sample (participants, mean age, male proportion, ICG composition)	Intervention Duration	Retention	Outcomes
Kajiyama et al. (2018) [12],<br>pilot study; pre-post,<br>USA	N=25, mean age 57.4,<br>24% male,<br>20% spouse; 64% children; 16% others	4 weeks	76.00%	stress, depression, caregiver knowledge
Kales et al. (2018) [13],<br>2-site pilot RCT; pre-post,<br>USA	N=57, mean age= 65.9, 24.56% male,<br>44% spouse; others 56%	1 month	96.50%	Caregiver distress, caregiver stress, burden, negative communication, relationship closeness, PLWD behavioral frequency, severity, and total behavioral score
Wijma et al. (2018) [14],<br>pilot; pre-post,<br>Netherlands	N=42, mean age= 55.1,<br>26.2% male,<br>29% spouse; 62% children; 10% other	3 weeks	83.33%	Person-centeredness (PC), empathy, stress, competence, and quality of the relationship.
Boots et al. (2017) [15],<br>RCT; pre-post,<br>Netherlands	N=49, mean age 69.6,<br>28.6% male,<br>98% spouse	NA	61.25%	process evaluation: sampling quality, intervention quality
Griffiths et al. (2015) [16],<br>pilot study; pre-post; convenient sampling,<br>USA	N=30, mean age 66,<br>3% male,<br>73% spouse; 23% children; 3% others	6 weeks	73.33%	Caregiver burden, anxiety, depressive symptoms, behavioral and psychological symptoms, caregiving competence
Núñez-Naveira et al. (2016) [17],<br>pilot RCT; pre-post,<br>Denmark; Portland; Spain	N=77, mean age not mentioned,<br>36.1 % male,<br>ICG composition not mentioned	NA	79.22%	Depression, perceived competence, satisfaction with caring experiences
Blom et al. (2015) [18],<br>RCT; pre-post,<br>Netherlands	N=251, mean age= 75.9,<br>39.6% male,<br>spouses 58.4%; children (in-law) 39.6%	6 months	69.72%	Depression, anxiety, burden, distress, competence, mastery
Finkel et al. (2015) [19],<br>RCT; pre-post,<br>USA	N=46, mean age 64.6,<br>Male percentage not mentioned,<br>44% spouse; 53% children	6 months	76.00%	Depression, caregiver burden, caregiver's health care behavior, caregiver's self-care, social support, patient’s problem behavior
Hattink, Bart et al. (2015) [20],<br>RCT; pre-post;<br>Netherlands and United Kingdom (UK)	N=142, mean age 52.9-54.7,<br>28.8% male,<br>50.7% informal caregivers; 16.9% volunteers; 32.4% professional caregivers	2-4 months	58.45%	Knowledge on dementia, attitudes regarding dementia. empathy, quality of life, burden, and sense of competence.<br>Intervention usefulness and user friendliness
Pot et al. (2015) [21],<br>secondary data-analysis from a pragmatic RCT,<br>Netherlands	N=149, mean age 61.5,<br>30% male,<br>60% spouse; 40% other family members or friends	4 weeks	44.30%	Cognitive decline of the people with dementia, psychological distress in caregivers
McKechnie et al., (2014) [22],<br>mixed method study; pre–post;<br>UK	N=128, mean age 56,<br>18% male,<br>31.9% partner; 56.3% children; 9.2% Other family members; 2.5% Other	12 weeks	NA	Depression, anxiety

Study, Design, Location	Sample (participants, mean age, male proportion, ICG composition)	Intervention Duration	Retention	Outcomes
O'Connor et al. (2014) [23],<br>feasibility pilot study; pre-post,<br>USA	N=10, mean age 60.86,<br>0% male,<br>43% spouse; 71% children; 14% siblings	8 weeks	70.00%	Loneliness, depression, caregiver burden, perceived stress in caregivers
Pagán-Ortiz et al. (2014) [24],<br>quasi-experiment; two-group design; pre-post;<br>Puerto Rico; Mexico; and Massachusetts	N=40, mean age not mentioned,<br>39 % male,<br>ICG composition not mentioned	3 months	80.00%	Self-efficacy, caregiver burden, caregiver distress
Bass et al. (2013) [25],<br>matched intervention and comparison sites with pre-post measures,<br>USA	N=486, mean age 69.1,<br>male percentage not mentioned,<br>72.6% spouse, others not mentioned	12 months	66.70%	Unmet needs, caregiver strains (role captivity, physical health strain,<br>and relationship strain), Depression,<br>support resources (number of informal helpers, use of caregiver support services)
Kajiyamaa et al. (2013) [26],<br>2 arm RCT; pre-post,<br>USA	N=150, mean age 56.12, 16% male,<br>54% spouse or partner; 35% children; 11% others	3 months	68.70%	Perceived stress, level of bother due to behaviors, depressive symptoms, perceived quality of life
Lorig et al. (2013) [27],<br>pilot; one group; pre-post,<br>USA	N=60, mean age 57.2,<br>18.3% male,<br>62% spouse (Others not mentioned)	6 weeks	75.00%	Caregiver burden, depression, pain, stress, self-efficacy health behaviors, social support
Marziali (2011) [28],<br>quasi experimental study; pre-post;<br>multisite; bilingual;<br>Canada	N=91, mean age= 65.51, 28% male,<br>74% spouse, 26% children (mostly daughters)	6 months	NA	Caregiver health, social support, caregiving distress, depress symptom
Lewis et al. (2010) [29],<br>mixed-method formative evaluation study,<br>USA	N=63, mean age 55,<br>15% male,<br>Other not mentioned	4 months	74.60%	Intervention feasibility and usefulness
Chiu et al. (2009) [30],<br>mixed method design; pre-post, Canada	N=35, mean age unknown,<br>32% male,<br>18% spouse or partner; 82% children	NA	80.00%	Caregiver burden, depression, perceived overall health, perceived social support, patient functional level, perceived competence, knowledge
Beauchamp et al. (2005) [31],<br>RCT; pre-post,<br>USA	N=329, mean age 46.9,<br>27% male,<br>7% spouse or partner; 67% children; 23% other family members; 3% non-relative	1 month	93.30%	Caregiver strain, caregiver gain, depression, anxiety
Eisdorfer et al. (2003) [32],<br>RCT; pre-post;<br>USA	N=225, mean age 69,<br>25 % male,<br>65% spouse; 27% children; and 8% other relatives	6 months	65.30%	Depressive symptoms, well-being, caregiver burden/upsetness, satisfaction with social support, self-reported health

References

1. Shaw CA, Williams KN, Lee RH, Coleman CK. Cost-effectiveness of a telehealth intervention for in-home dementia care support: Findings from the FamTechCare clinical trial. *Res Nurs Health*. 2021;44(1):60-70. doi:10.1002/nur.22076
2. Williams KN, Shaw CA, Perkhounkova Y, Hein M, Coleman CK. Satisfaction, utilization, and feasibility of a telehealth intervention for in-home dementia care support: A mixed methods study. *Dement Lond Engl*. 2021;20(5):1565-1585. doi:10.1177/1471301220957905
3. Bruinsma J, Peetoom K, Boots L, et al. Tailoring the web-based "Partner in Balance" intervention to support spouses of persons with frontotemporal dementia. *Internet Interv*. 2021;26:100442. doi:10.1016/j.invent.2021.100442
4. Baruah U, Varghese M, Loganathan S, et al. Feasibility and preliminary effectiveness of an online training and support program for caregivers of people with dementia in India: a randomized controlled trial. *Int J Geriatr Psychiatry*. 2021;36(4):606-617.
5. James TA, James D, Larkey LK. Heart-focused breathing and perceptions of burden in Alzheimer's caregivers: An online randomized controlled pilot study. *Geriatr Nur (Lond)*. 2021;42(2):397-404.
6. Fossey J, Charlesworth G, Fowler JA, et al. Online education and cognitive behavior therapy improve dementia caregivers' mental health: A randomized trial. *J Am Med Dir Assoc*. 2021;22(7):1403-1409.
7. Romero-Mas M, Ramon-Aribau A, Souza DLB de, Cox AM, Gómez-Zúñiga B. Improving the Quality of Life of Family Caregivers of People with Alzheimer's Disease through Virtual Communities of Practice: A Quasiexperimental Study. *Int J Alzheimer's Dis*. 2021;2021.
8. Verkade PJ, Twisk JW, Verkaik R, Marco M, van Meijel B, Francke AL. Online self-management support for family caregivers dealing with behavior changes in relatives with dementia (Part 2): Randomized controlled trial. *J Med Internet Res*. 2020;22(2):e13001.
9. Gustafson Jr DH, Gustafson Sr DH, Cody OJ, Chih MY, Johnston DC, Asthana S. Pilot test of a computer-based system to help family caregivers of dementia patients. *J Alzheimers Dis*. 2019;70(2):541-552.
10. Duggleby W, Jovel Ruiz K, Ploeg J, et al. Mixed-methods single-arm repeated measures study evaluating the feasibility of a web-based intervention to support family carers of persons with dementia in long-term care facilities. *Pilot Feasibility Stud*. 2018;4(1):1-12.
11. Duggleby W, Ploeg J, McAiney C, et al. Web-based intervention for family carers of persons with dementia and multiple chronic conditions (My Tools 4 Care): pragmatic randomized controlled trial. *J Med Internet Res*. 2018;20(6):e10484.
12. Kajiyama B, Fernandez G, Carter EA, Humber MB, Thompson LW. Helping Hispanic dementia caregivers cope with stress using technology-based resources. *Clin Gerontol*. 2018;41(3):209-216.
13. Kales HC, Gitlin LN, Stanislawski B, et al. Effect of the WeCareAdvisor™ on family caregiver outcomes in dementia: a pilot randomized controlled trial. *BMC Geriatr*. 2018;18(1):1-12.
14. Wijma EM, Veerbeek MA, Prins M, Pot AM, Willemse BM. A virtual reality intervention to improve the understanding and empathy for people with dementia in informal caregivers: results of a pilot study. *Aging Ment Health*. 2018;22(9):1121-1129.
15. Boots LM, de Vugt ME, Smeets CM, Kempen GI, Verhey FR. Implementation of the blended care self-management program for caregivers of people with early-stage dementia (Partner in Balance): process evaluation of a randomized controlled trial. *J Med Internet Res*. 2017;19(12):e423.
16. Griffiths PC, Whitney MK, Kovaleva M, Hepburn K. Development and implementation of tele-savvy for dementia caregivers: a department of veterans affairs clinical demonstration project. *The Gerontologist*. 2016;56(1):145-154.
17. Núñez-Naveira L, Alonso-Búa B, de Labra C, et al. UnderstAID, an ICT platform to help informal caregivers of people with dementia: a pilot randomized controlled study. *BioMed Res Int*. 2016;2016.
18. Blom MM, Zarit SH, Groot Zwaaftink RB, Cuijpers P, Pot AM. Effectiveness of an Internet intervention for family caregivers of people with dementia: results of a randomized controlled trial. *PloS One*. 2015;10(2):e0116622.
19. Finkel S, Czaja S, Schulz R, Martinovich Z, Harris C, Pezzuto D. E-Care: A Telecommunications Technology Intervention for Family Caregivers of Dementia Patients. *Am J Geriatr Psychiatry Off J Am Assoc Geriatr Psychiatry*. 2007;15:443-448. doi:10.1097/JGP.0b013e3180437d87
20. Hattink B, Meiland F, van der Roest H, et al. Web-based STAR E-learning course increases empathy and understanding in dementia caregivers: results from a randomized controlled trial in the Netherlands and the United Kingdom. *J Med Internet Res*. 2015;17(10):e241.
21. Pot AM, Blom MM, Willemse BM. Acceptability of a guided self-help Internet intervention for family caregivers: mastery over dementia. *Int Psychogeriatr*. 2015;27(8):1343-1354.

22. McKechnie V, Barker C, Stott J. The effectiveness of an Internet support forum for carers of people with dementia: a pre-post cohort study. *J Med Internet Res*. 2014;16(2):e68.
23. O'Connor MF, Arizmendi BJ, Kaszniak AW. Virtually supportive: a feasibility pilot study of an online support group for dementia caregivers in a 3D virtual environment. *J Aging Stud*. 2014;30:87-93.
24. Pagán-Ortiz ME, Cortés DE, Rudloff N, Weitzman P, Levkoff S. Use of an online community to provide support to caregivers of people with dementia. *J Gerontol Soc Work*. 2014;57(6-7):694-709.
25. Bass DM, Judge KS, Lynn Snow A, et al. Caregiver outcomes of partners in dementia care: effect of a care coordination program for veterans with dementia and their family members and friends. *J Am Geriatr Soc*. 2013;61(8):1377-1386.
26. Kajiyama B, Thompson LW, Eto-Iwase T, et al. Exploring the effectiveness of an internet-based program for reducing caregiver distress using the iCare Stress Management e-Training Program. *Aging Ment Health*. 2013;17(5):544-554.
27. Lorig K, Thompson-Gallagher D, Traylor L, et al. Building better caregivers: a pilot online support workshop for family caregivers of cognitively impaired adults. *J Appl Gerontol*. 2012;31(3):423-437.
28. Marziali E, Garcia LJ. Dementia caregivers' responses to 2 internet-based intervention programs. *Am J Alzheimers Dis Dementias*®. 2011;26(1):36-43.
29. Lewis ML, Hobday JV, Hepburn KW. Internet-based program for dementia caregivers. *Am J Alzheimers Dis Dementias*®. 2010;25(8):674-679.
30. Chiu T, Marziali E, Colantonio A, et al. Internet-based caregiver support for Chinese Canadians taking care of a family member with Alzheimer disease and related dementia. *Can J Aging Rev Can Vieil*. 2009;28(4):323-336.
31. Beauchamp N, Irvine AB, Seeley J, Johnson B. Worksite-based internet multimedia program for family caregivers of persons with dementia. *The Gerontologist*. 2005;45(6):793-801.
32. Eisdorfer C, Czaja SJ, Loewenstein DA, et al. The effect of a family therapy and technology-based intervention on caregiver depression. *The Gerontologist*. 2003;43(4):521-53
