# Supplementary material for: Behavioral Change Factors and Retention in Web-Based Interventions for Informal Caregivers of People Living With Dementia: Scoping Review
Source: J Med Internet Res. 2022 Jul 7;24(7):e38595. doi: 10.2196/38595 (PMC9305400; doi:10.2196/38595)
Supplement: Multimedia Appendix 3 [file jmir_v24i7e38595_app3.docx]

**Multimedia Appendix 3:** The behavioral change factors (theory, techniques, and agents) identified in each study.

| Study | Intervention | Theory | BCT^a^v1 clusters and individual BCTs | BCTv1 cluster count | Individual BCT count | Individual BCA^b^; BCA domain |
| --- | --- | --- | --- | --- | --- | --- |
| Shaw et al, 2021 [78] | FamTechCare | N/A^c^ | 9. Comparison of outcomes   - 9.1 Credible source   12. Antecedents   - 12.5 Adding objects to the environment | 2 | 2 | N/A |
| Williams et al, 2021 [79] | FamTechCare | N/A | 2. Feedback and monitoring   - 2.2 Feedback on behavior - 2.3 Self-monitoring behavior   4. Shaping knowledge   - 4.1 Instructions on how to perform a behavior   9. Comparison of outcomes   - 9.1 Credible source   12. Antecedents   - 12.5 Adding objects to the environment | 4 | 5 | N/A |
| Bruinsma et al, 2021 [80] | PiB^d^ | Stress and Coping paradigm | 1. Goal and planning   - 1.1 Goal planning (behavior) - 1.2 Problem solving - 1.4 Action planning   2. Feedback and monitoring   - 2.3 Self-monitoring - behavior   3. Social support   - 3.1 Social support (unspecified) - 3.3 Social support (emotional)   4. Shaping knowledge   - 4.1 Instructions on how to perform a behavior - 4.2 information about antecedents   6. Comparison of behavior   - 6.1 demonstration of the behavior - 6.2 social comparison   9. Comparison of outcomes   - 9.1 Credible source   13. Identity   - 13.2 Framing or reframing | 7 | 12 | Self-efficacy, stress, and burden; stress reactivity and self-regulation |
| Baruah et al, 2021 [81] | iSupport | BCT, Psychoeducation | 1. Goal and planning   - 1.2 Problem solving   2. Feedback and monitoring   - 2.2 Feedback on behavior   4. Shaping knowledge   - 4.1 Instructions on how to perform a behavior - 4.2 Information about antecedents   6. Comparison of behavior   - 6.1 Demonstration of the behavior   12. Antecedents   - 12.6 Body changes   13. Identity   - 13.2 Framing or reframing | 6 | 7 | Burden, depression, self-efficacy, and mastery; self-regulation and stress reactivity |
| James et al, 2021 [82] | Web-based intervention on heart-focused breathing | N/A | 4. Shaping knowledge   - 4.2 Information about antecedents   6. Comparison of behavior   - 6.1 Demonstration of the behavior   12. Antecedents   - 12.6 Body changes   13. Identity   - 13.2 Framing or reframing | 4 | 4 | Self-compassion and caregiver burden; self-regulation and stress reactivity |
| Fossey et al, 2021 [83] | Caring for Me and You | CBT^e^, Psychoeducation | 1. Goal and planning   - 1.1 Goal planning (behavior) - 1.4 Action planning   3. Social support   - 3.1 Social support (unspecified)   4. Shaping knowledge   - 4.1 Instructions on how to perform a behavior   6. Comparison of behavior   - 6.1 Demonstration of the behavior - 6.2 Social comparison   9. Comparison of outcomes   - 9.3 Comparative imagining of future outcomes   13. Identity   - 13.2 Framing or reframing | 6 | 8 | Overall mood, caregiving stress, and caregiver mastery; self-regulation and stress reactivity |
| Romero-Mas et al, 2021 [84] | Mobile app: Virtual Community of Practice | CoP^f^ theory | 3. Social support   - 3.1 Social support (unspecified) - 3.3 Social support (emotional)   4. Shaping knowledge   - 4.1 Instructions on how to perform a behavior   9. Comparison of outcomes   - 9.1 Credible source | 3 | 4 | N/A |
| Verkade et al, 2020 [57] | Web-based self-management support intervention (major, medium, or minor intervention) | N/A | 2. Feedback and monitoring   - 2.2 Feedback on behavior   4. Shaping knowledge   - 4.1 Instruction on how to perform   9. Comparison of outcomes   - 9.1 Credible source | 3 | 3 | Self-efficacy; self-regulation |
| Gustafson et al, 2019 [52] | Dementia–Comprehensive Health Enhancement Support System | N/A | 1. Goal and planning   - 1.2 Problem solving - 1.4 Action planning   2. Feedback and monitoring   - 2.2 Feedback on behavior - 2.4 Self-monitoring of outcomes of behavior   3. Social support   - 3.1 Social support (unspecified) - 3.2 Social support (practical)   4. Shaping knowledge   - 4.1 Instructions on how to perform a behavior   5. Natural consequences   - 5.6 Information about emotional consequences   6. Comparison of behavior   - 6.2 Social comparison   9. Comparison of outcomes   - 9.1 Credible source   12. Antecedents   - 12.2 Restructuring the social environment - 12.5 Adding objects to the environment - 12.6 Body changes   15. Self-belief   - 15.1 Verbal persuasion about capability | 9 | 14 | Coping competence, caregiver burden, or social support; self-regulation, stress reactivity, or interpersonal |
| Duggleby et al, 2018 [46] | MT4C^g^ | Transition theory | 4. Shaping knowledge   - 4.1. Instruction on how to perform the behavior   13. Identity   - 13.2 Framing or reframing | 2 | 2 | Self-efficacy; self-regulation |
| Duggleby et al, 2018 [45] | MT4C-In Care | Transition theory | 4. Shaping knowledge   - 4.1 Instructions on how to perform a behavior - 4.2 Information about antecedents   6. Comparison of behavior   - 6.2 Social comparison   9. Comparison of outcomes   - 9.1 Credible source | 3 | 4 | Self-efficacy; self-regulation |
| Kajiyama et al, 2018 [43] | Webnovela Mirela | CBT | 4. Shaping knowledge   - 4.1 Instruction on how to perform a behavior   6. Comparison of behavior   - 6.1 Demonstration of the behavior | 2 | 2 | Perceived stress; stress reactivity |
| Kales et al, 2018 [53] | WeCareAdvisor | N/A | 1. Goal and planning   - 1.2 Problem solving - 1.4 Action planning   3. Social support   - 3.1 Social support (unspecified) - 3.3 Social support (emotional)   4. Shaping knowledge   - 4.1 Instructions on how to perform a behavior | 3 | 5 | Stress, burden, and confidence; stress reactivity, interpersonal, and self-regulation |
| Wijma et al, 2018 [59] | Into D’mentia | N/A | 4. Shaping knowledge   - 4.1 Instruction on how to perform a behavior   5. Natural consequences   - 5.3 Information about social and environmental consequences   6. Comparison of behavior   - 6.2 Social comparison | 3 | 3 | Empathy and perceived competence; self-regulation |
| Boots et al, 2017 [38] | PiB | Stress and Coping model | 1. Goal and planning   - 1.3 Goal setting - 1.6 Discrepancy between current behavior and goal - 1.7 Review outcome goals   2. Feedback and monitoring   - 2.2 Feedback on behavior - 2.3 Self-monitoring behavior   3. Social support   - 3.1 Social support (unspecified)   4. Shaping knowledge   - 4.1 Instruction on how to perform a behavior   9. Comparison of outcomes   - 9.1 Credible source | 5 | 8 | N/A |
| Griffiths et al, 2016 [51] | Tele-Savvy or adapted from Savvy Caregiver Program | SCT^h^, Stress and Coping model | 4. Shaping knowledge   - 4.1 Instructions on how to perform a behavior   6. Comparison of behavior   - 6.1 Demonstration of the behavior   9. Comparison of outcomes   - 9.1 Credible source   12. Antecedents   - 12.6 Body changes   15. Self-belief   - 15.1 Verbal persuasion about capability | 5 | 5 | Burden and caregiving competence; stress reactivity and self-regulation |
| Núñez-Naveira et al, 2016 [58] | UnderstAID | N/A | 1. Goal and planning   - 1.2 Problem solving   3. Social Support   - 3.1 Social support (unspecified)   4. Shaping knowledge   - 4.1 Instruction on how to perform a behavior   6. Comparison of behavior   - 6.1 demonstration of the behavior   7. Association   - 7.1 Prompts or cues   8. Repetition and substitution   - 8.7 Graded tasks | 6 | 6 | Caregiving competencies; self-regulation |
| Blom et al, 2015 [41] | MoD^i^ | CBT, Psychoeducation | 1. Goal and planning   - 1.2 Problem solving   2. Feedback and monitoring   - 2.1. Monitoring of behavior by others without feedback - 2.2 Feedback on behavior   6. Comparison of behavior   - 6.1 Demonstration of the behavior   7. Associations   - 7.1 Prompt and cues   8. Repetition and substitution   - 8.1 Behavioral practice or rehearsal   9. Comparison of outcomes   - 9.1 Credible source   12. Antecedents   - 12.6 Body changes   13. Identity   - 13.2 Framing or reframing   15. Self-belief   - 15.1 Verbal persuasion about capability | 9 | 10 | Burden, mastery, and distress; self-regulation and stress reactivity |
| Finkel et al, 2015 [50] | e-Care or adapted from Resources for Enhancing Alzheimer’s Caregiver Health program | N/A | 3. Social support   - 3.1 Social support (unspecified)   4. Shaping knowledge   - 4.1 Instructions on how to perform a behavior   9. Comparison of outcomes   - 9.1 Credible source | 3 | 3 | Burden and support; stress reactivity and interpersonal |
| Hattink et al, 2015 [47] | European Skills Training and Reskilling | Adaption-Coping model | 1. Goals and planning   - 1.2 Problem solving   2. Feedback and monitoring   - 2.7 Feedback on outcome or outcomes of behavior   3. Social support   - 3.1 Social support (unspecified)   4. Shaping knowledge   - 4.1. Instruction on how to perform the behavior - 4.2 Information about antecedents.   5. Natural consequences   - 5.1 Information about health consequences - 5.6 Information about emotional consequences   6. Comparison of behavior   - 6.1 Demonstration of the behavior   8. Repetition and substitution   - 8.1 Behavior practice or rehearsal   9. Comparison of outcomes   - 9.1 Credible source   13. Identity   - 13.2 Framing or reframing | 7 | 11 | Burden and stress; self-regulation and stress reactivity |
| Pot et al, 2015 [44] | MoD | CBT, Psychoeducation | 1. Goals and planning   - 1.2 Problem solving   3. Social support   - 3.1 Social support (unspecified)   4. Shaping knowledge   - 4.1 Instruction on how to perform a behavior - 4.2 Information about antecedents   6. Comparison of behavior   - 6.1 Demonstration of the behavior   12. Antecedents   - 12.1 Restructuring the physical environment - 12.6 Body changes   13. Identity   - 13.2 Framing or reframing   15. Self-belief   - 15.4 Self-talking | 7 | 9 | Caregiver burden; stress reactivity |
| McKechnie et al, 2014 [77] | Talking Point’s web-based forum | N/A | 3. Social support   - 3.1 Social support (unspecified) | 1 | 1 | N/A |
| O’Connor et al, 2014 [55] | Virtual Reality web-based caregiver support group | N/A | 3. Social support   - 3.1 Social support (unspecified)   4. Shaping knowledge   - 4.1. Instruction on how to perform the behavior   9. Comparison of outcomes   - 9.1. Credible source   12. Antecedents   - 12.1. Restructuring the physical environment   13. Identity   - 13.2 Framing or reframing | 5 | 5 | Loneliness, burden, and stress; stress reactivity and interpersonal |
| Pagán-Ortiz et al, 2014 [56] | Caregiver, take care of yourself | N/A | 1. Goal and planning   - 1.2 Problem solving   3. Social support   - 3.1 Social support (unspecified)   4. Shaping knowledge   - 4.1 Instruction on how to perform a behavior   6. Comparison of behavior   - 6.1 Demonstration of the behavior | 4 | 4 | Self-efficacy and social support; self-regulation and interpersonal |
| Bass et al, 2013 [36] | Partners in Dementia Care or adapted version of BRI^j^ Care Consultation | SPM^k^ | 1. Goal and planning   - 1.2 Problem solving - 1.4 Action planning   2. Feedback and monitoring   - 2.1 Monitoring of behavior by others without feedback   3. Social support   - 3.3 Social support (emotional)   9. Comparison of outcomes   - 9.1 Credible source | 4 | 5 | Caregiving strain and support; stress reactivity and interpersonal |
| Kajiyamaa et al, 2013 [42] | iCare Condition or adapted from a psychoeducational program called “Coping with Caregiving” | CBT, TBR^l^, Psychoeducation | 1. Goal and planning   - 1.2 Problem solving - 1.4 Action planning   2. Feedback and monitoring   - 2.3 Self-monitoring behavior   4. Shaping knowledge   - 4.1 Instructions on how to perform a behavior - 4.2 Information about antecedents   6. Comparison of behavior   - 6.1 Demonstration of the behavior   11. Regulation   - 11.2 Reduce negative emotions   12. Antecedents   - 12.6 Body changes   13. Identity   - 13.2 Framing or reframing | 7 | 9 | Perceived stress; stress reactivity |
| Lorig et al, 2013 [54] | Building Better Caregiver | N/A | 1. Goals and planning   - 1.2 Problem solving - 1.4 Action Planning   2. Feedback and monitoring   - 2.1. Monitoring of behavior by others without feedback   3. Social support   - 3.1 Social support (unspecified)   4. Shaping knowledge   - 4.1 Instruction on how to perform a behavior   7. Associations   - 7.1 Prompts or cues | 5 | 6 | Self-efficacy, caregiver burden, and stress; self-regulation and stress reactivity |
| Marziali and Garcia, 2011 [40] | Online Chat Group + Caring for Me WebsiteOnline Video Group | Stress and Coping model | 1. Goal and planning   - 1.2 Problem solving   2. Feedback and monitoring   - 2.1 Monitoring of behavior by others without feedback   3. Social support   - 3.1 Social support (unspecified) - 3.3 Social support (emotional)   4. Shaping knowledge   - 4.1 Instructions on how to perform a behavior   6. Comparison of behavior   - 6.1 Demonstration of the behavior - 6.2 Social comparison   9. Comparison of outcomes   - 9.1 Credible source | 6 | 8 | Perceived Social Support, self-efficacy, and distress; interpersonal, stress reactivity, and self-regulation |
| Lewis et al, 2010 [39] | Internet-Based Savvy Caregiver | Stress and Coping model | 1. Goal and planning   - 1.2 Problem solving - 1.4 Action planning   4. Shaping knowledge   - 4.1 Instructions on how to perform a behavior - 4.2 Information about antecedents   6. Comparison of behavior   - 6.1 Demonstration of the behavior   9. Comparison of outcomes   - 9.1 Credible source | 4 | 6 | N/A |
| Chiu et al, 2009 [48] | Internet-based Caregiver Support Service | N/A | 3. Social support   - 3.1 Social support (unspecified) - 3.3 Social support (emotional)   4. Shaping knowledge   - 4.1 Instructions on how to perform a behavior   9. Comparison of outcomes   - 9.1 Credible source | 3 | 4 | Perceived burden, support and competencies; stress reactivity, interpersonal, and self-regulation |
| Beauchamp et al, 2005 [37] | Caregiver’s friend: dealing with dementia | Stress and Coping model | 1. Goal and planning   - 1.2 Problem solving   3. Social support   - 3.1 Social support (unspecified)   4. Shaping knowledge   - 4.1 Instruction on how to perform a behavior   6. Comparison of behavior   - 6.1 demonstration of the behavior   10. Reward and threat   - 10.3 Nonspecific reward | 5 | 5 | Stress, coping skills, and self-efficacy; self-regulation, stress reactivity, and interpersonal |
| Eisdorfer et al, 2003 [49] | SET^m^, SET+ Computer–Telephone Integrated System | N/A | 2. Feedback and monitoring   - 2.2 Feedback on behavior   3. Social support   - 3.1 Social support (unspecified)   7. Associations   - 7.1 Prompt and cues   8. Repetition and substitution   - 8.1 Behavior practice or rehearsal   9. Comparison of outcomes   - 9.1 Credible source | 5 | 5 | Social support and burden; stress reactivity and interpersonal |

^a^BCT: behavior change technique.

^b^BCA: behavior change agent.

^c^N/A: not applicable.

^d^PiB: Partner in Balance

^e^CBT: cognitive behavior therapy.

^f^CoP: communities of practice.

^g^MT4C: My Tools 4 Care.

^h^SCT: social cognitive theory.

^i^MoD: Mastery over Dementia.

^j^BRI: Benjamin Rose Institute on Aging

^k^SPM: stress process model.

^l^TBR: trigger behavior response.

^m^SET: Structural Ecosystems Therapy.
